# Cytoprotective Nrf2 Pathway Is Induced In Chronically Txnrd 1-Deficient Hepatocytes

**DOI:** 10.1371/journal.pone.0006158

**Published:** 2009-07-07

**Authors:** Elena S. Suvorova, Olivier Lucas, Carla M. Weisend, MaryClare F. Rollins, Gary F. Merrill, Mario R. Capecchi, Edward E. Schmidt

**Affiliations:** 1 Veterinary Molecular Biology, Montana State University, Bozeman, Montana, United States of America; 2 Biochemistry and Biophysics, Oregon State University, Corvallis, Oregon, United States of America; 3 Howard Hughes Medical Institute (HHMI), University of Utah, Salt Lake City, Utah, United States of America; 4 Center for Reproductive Biology, Washington State University, Pullman, Washington, United States of America; Roswell Park Cancer Institute, United States of America

## Abstract

**Background:**

Metabolically active cells require robust mechanisms to combat oxidative stress. The cytoplasmic thioredoxin reductase/thioredoxin (Txnrd1/Txn1) system maintains reduced protein dithiols and provides electrons to some cellular reductases, including peroxiredoxins.

**Principal Findings:**

Here we generated mice in which the *txnrd1* gene, encoding Txnrd1, was specifically disrupted in all parenchymal hepatocytes. Txnrd1-deficient livers exhibited a transcriptome response in which 56 mRNAs were induced and 12 were repressed. Based on the global hybridization profile, this represented only 0.3% of the liver transcriptome. Since most liver mRNAs were unaffected, compensatory responses were evidently effective. Nuclear pre-mRNA levels indicated the response was transcriptional. Twenty-one of the induced genes contained known antioxidant response elements (AREs), which are binding sites for the oxidative and chemical stress-induced transcription factor Nrf2. Txnrd1-deficient livers showed increased accumulation of nuclear Nrf2 protein and chromatin immunoprecipitation on the endogenous *nqo1* and *aox1* promoters in fibroblasts indicated that Txnrd1 ablation triggered *in vivo* assembly of Nrf2 on each.

**Conclusions:**

Chronic deletion of Txnrd1 results in induction of the Nrf2 pathway, which contributes to an effective compensatory response.

## Introduction

Liver metabolic pathways support organismal homeostasis and detoxify xenobiotics [1]. As a result of these metabolic functions, liver encounters both continuous and intermittent oxidative challenges. To counteract endogenous reactive oxygen species (ROS) production, hepatocytes have abundant antioxidant enzymes, including thioredoxin reductases (Txnrd), glutathione reductase (GSR), superoxide dismutase, catalase, peroxiredoxins (Prx), and glutathione peroxidases (Gpx), and they have high levels of electron carriers, such as thioredoxins (Txn), glutaredoxins (Grx), and glutathione (GSH) [2] to transfer reducing potential to other reductases. Thiol-containing molecules are particularly sensitive to oxidation. The Txnrd/Txn system plays a major role in maintaining or restoring thiols [3] and cytoplasmic Txnrd1/Txn1-dependent Prxs actively detoxify hydrogen peroxide [4], [5].

In addition to these constitutive antioxidant systems, hepatocytes have inducible oxidative stress-response pathways [2]. For example, following transient oxidative or chemical challenge, hepatocytes induce the Nrf2 pathway [6], [7]. Nrf2, a bZIP family transcription factor, activates expression of many genes involved in cytoprotective responses [8], [9]. In unstressed cells, Nrf2 interacts with the ubiquitination adapter protein Keap1, which targets Nrf2 for proteosomal degradation [10]. Oxidative challenge induces stabilization of Nrf2 in the nucleus, where it heterodimerizes with the ubiquitous bZIP protein Maf and binds to antioxidant responsive elements (ARE) in regulatory regions of Nrf2-response genes [8], [10]. Genetic disruption of Nrf2 does not cause chronic oxidative stress, but it renders cells more susceptible to oxidative challenge [6], [11], [12]. Hepatocytic disruption of Keap1 activates the Nrf2 pathway and results in increased resistance to oxidative challenges [9]. Thus, the Nrf2 pathway provides a rapid feedback-triggered mechanism of countering transiently severe oxidative challenges.

Txnrds are ubiquitous flavin-containing NADPH-dependent enzymes that restore oxidized Txn to a reduced dithiol state [13]. Mammals have three Txnrd proteins: cytoplasmic Txnrd1, mitochondrial Txnrd2, and the testis-specific Txnrd3 [14]. Disruption of the *txnrd1* gene is lethal in embryogenesis [15] or during fetal organogenesis [16], depending on the design of the mutant allele. Because the Txnrd1/Txn1 pathway participates in constitutive maintenance of the redox state of hepatocytes, one might predict that disruption of this pathway in liver would result in chronic oxidative stress. However, Txnrd1-deficient hepatocytes are long-term viable and do not exhibit hallmark signs of chronic oxidative stress (ESS, GFM, and EES, unpublished data; and see below). In the current study, transcriptome analyses showed activation of cytoprotective mRNAs in Txnrd1-deficient liver. Many of these were encoded by Nrf2-response genes. These results suggest that oxidative/chemical stress response pathways are able to compensate for chronic defects in the constitutive antioxidant pathways.

## Results

### Establishment of Mice Bearing *txnrd1^−/−^* Hepatocytes

We previously reported generation of a mouse line bearing a conditional-null *txnrd1* allele, entitled *txnrd1^cond^*, that converts to a true null (*txnrd1^−^*) upon expression of Cre [15]. To examine a role of Txnrd1 in the liver, we crossed these to mice bearing 1 or 2 copies of the *albCre* transgene (denoted *albCre^1^* or *albCre^2^*, respectively), in which Cre recombinase is expressed exclusively and constitutively by hepatocytes [17]. Further husbandry yielded *txnrd1^cond/−^*;*albCre^1^* and *txnrd1^cond/cond^*;*albCre^1^* mice, in which all hepatocytes should be *txnrd1^−/−^*. Although Txnrd1 is required for embryonic development [15], [16], these mice were fully viable for >1 year. *txnrd1^cond/+^*;*albCre^0^* mice (no *albCre* transgene) served as controls.

**Figure 1:**
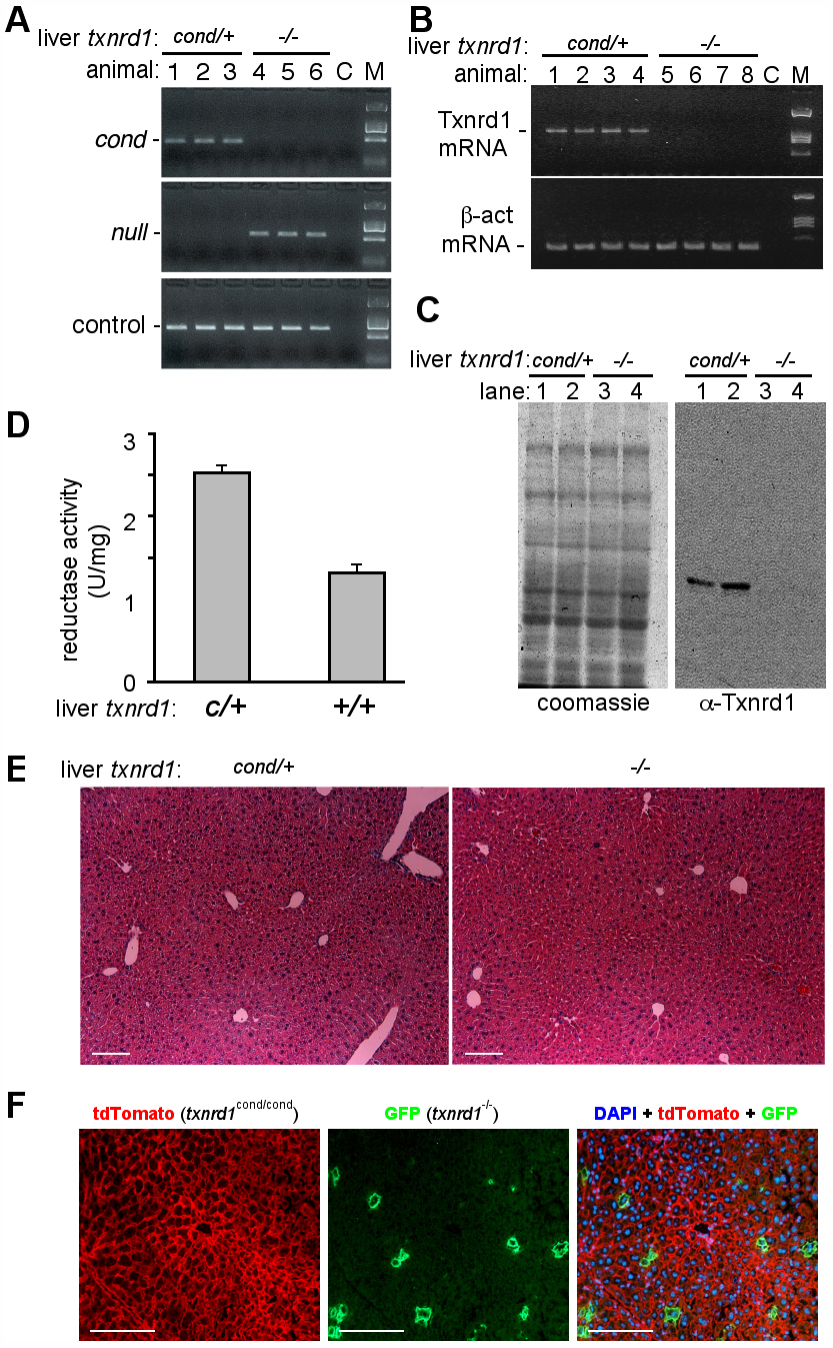
Allelic conversion, Txnrd1 mRNA expression, and histology of livers from *txnrd1^cond/cond^*;*albCre^1^* and *txnrd1^cond/−^*;*albCre^1^* mice. (A) Allelic conversion. Shown are PCR reactions on genomic DNA from livers of three control mice, genotype *txnrd1^cond/+^*;*albCre^0^* (animals 1–3; liver *txnrd1^cond/+^*), and three experimental mice, genotype *txnrd1^cond/−^*;*albCre^1^* (animals 4–6; liver *txnrd1^−/−^*). PCR reactions for the *txnrd1^cond^* allele (top panel), *txnrd1^−^* allele (middle panel), and a control genomic DNA sequence (*aox1* promoter; lower panel) are shown. C, no template control. M, molecular size markers. (B) Txnrd1 mRNA expression. RT-PCR reactions used oligo(dT)-primed cDNA of total RNA harvested from four *txnrd1^cond/+^*;*albCre^0^* adults (lanes 1–4; liver *txnrd1^cond/+^*) and four *txnrd1^cond/−^*;*albCre^1^* adults (lanes 5–8; liver *txnrd1^−/−^*). Abbreviations as in (A). (C) Txnrd1 protein expression. Shown is a coomassie-stained gel and western blot of total liver lysates from two *txnrd1^cond/+^*;*albCre^0^* adults (lanes 1, 2; hepatocytes *txnrd1^cond/+^*) and two *txnrd1^cond/−^*;*albCre^1^* adults (lanes 3, 4; hepatocytes *txnrd1^−/−^*) using affinity purified rabbit-anti-Txnrd1 antibody. M, molecular weight markers. (D) Combined reductase activity. Reduction of 5,5′-dithiobis(2-nitrobenzoic acid) by liver cytoplasmic extracts from *txnrd1^cond/+^*;*albCre^0^* (*cond/+*) or *txnrd1^cond/−^*;*albCre^1^* (*−/−*) adults. Depicted are averages and S.E.M. from four animals of each genotype. (E) Histology of young adult liver from *txnrd1^cond/+^*;*albCre^0^* (cond/+) and *txnrd1^cond/-^*;*albCre^1^* (−/−) mice. Mice were sacrificed at four months of age and livers were prepared for paraffin embedding. Sections were stained with H&E. Scale bars 100 nm. (F) Long-term survival of *txnrd1^−/−^* hepatocytes in mosaic livers. *txnrd1^cond/cond^*;*ROSA^mT-mG/+^* mice received ∼10^7^ PFU of AdCre I.V. at 2 months of age and livers were harvested six weeks later. Shown are fluorescence micrographs of DAPI-stained cryosections photographed under the red channel, green channel, or merged red + green + blue; red membranes are from cells in which *ROSA^mT-mG^* has not converted and green membranes are from cells in which *ROSA^mT-mG^* has been converted by Cre. Scale bars 100 nm.

The efficiency of allelic conversion and levels of functional Txnrd1 mRNA and protein were measured. Adult liver is comprised of ∼80% hepatocytes [18], which, due to their large size, constitute ∼95% of the mass of the organ [19]. Thus, 100% hepatocytic recombination on whole-liver assays will manifest as 80% allelic conversion or 95% reduction in mRNA or protein levels. Consistent with full conversion, livers from *txnrd1^cond/−^*;*albCre^1^* adults exhibited 80% allelic conversion (Fig. 1A) and a 20-fold reduction in levels of functional hepatic Txnrd1 mRNA and Txnrd1 protein (Figs. 1B, C).

Txnrd1-deficient livers exhibited diminished Txnrd enzyme activity (ESS, GFM, and EES, unpublished data). An enzymatic assay that measured the combined activities of all Txnrd and GSR enzymes showed a two-fold lower signal in *txnrd1^−/−^* as compared to control liver lysates (Fig. 1D), which was consistent with ablation of a major cellular reductase. However, mutant livers were histologically similar to control livers at four months of age (Fig. 1E).

To determine whether sustained normal histology resulted from persistence of mutant hepatocytes or from rapid turn-over of Txnrd1-deficient cells and replacement from a *txnrd1^cond/−^* progenitor/stem cell population, we generated mice with mosaic livers in which a subset of hepatocytes were converted to *txnrd1^−/−^* by a single intravenous inoculation with replication-defective adenovirus expressing Cre (AdCre) [20]. Because AdCre cannot replicate in mice and rapidly clears the system, allelic conversion only occurred during a short window of time following inoculation. To identify converted cells, we used the *ROSA^mT-mG^* indicator allele, which drives ubiquitous red fluorescence in all cells not exposed to Cre and green fluorescence in cells that have been exposed to Cre [21]. Control experiments showed tight correlation between the indicator allele and *txnrd1* allelic conversion (Fig. S1). Six weeks after AdCre administration, *txnrd1^−/−^* (green) hepatocytes persisted, indicating that they were not a rapidly turned-over population (Fig. 1F). Similar results were obtained using a Tamoxifen-inducible Cre expression system (Fig. S2 and not shown).

### Transcriptome Response to Txnrd1 Disruption

**Figure 2:**
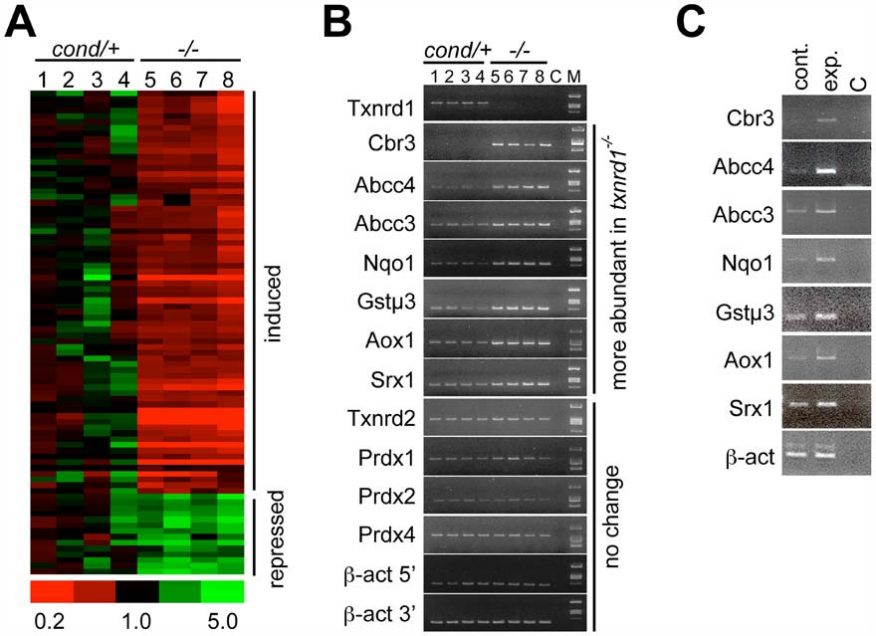
Transcriptome response to hepatocytic deletion of *txnrd1.* (A) Hierarchical clustering of mRNAs whose abundance differs between *txnrd1^cond/+^* and *txnrd1^−/−^* livers. Total RNA of livers harvested from four *txnrd1^cond/+^*;*albCre^0^* (*c/+*) and four *txnrd1^cond/−^*;*albCre^1^* (*−/−*) mice were analyzed on microarrays. Signal profiles revealed that 25,565 probe-sets (56.7% of total on array) gave a measurable average raw signal (≥50 units). Data were analyzed by GeneSpring; threshold values for differential mRNA abundance were established as ≥1.5-fold difference between average values, *p*≤0.05, and average raw signal strength ≥50 units. By these criteria, 84 probe-sets representing 68 different mRNAs were differentially abundant between *c/+* and −/− livers. A clustered heat diagram for these 84 probe-sets is depicted, with the scale at bottom. mRNA-specific data are listed in Table S1. (B) RT-PCR verification of sentinel mRNA levels. RT-PCR reactions were performed for the indicated mRNAs using primer sets listed in Table S2. mRNA abbreviations as in text; C, no template control; M, molecular size markers. (C) Nuclear pre-mRNA levels. Liver nuclei from control *txnrd1^cond/+^*;*albCre^0^* (cont.) and experimental *txnrd1^cond/−^*;*albCre^1^* (exp.) mice were purified by sedimentation through sucrose cushions and DNA-free RNA was prepared by sedimentation through CsCl cushions. Random-primed cDNA was prepared and RT-PCR analyses were performed using primer sets listed in Table S2. C, no template control.

The Txnrd1/Txn1 cycle provides electrons to many cellular redox pathways [22]. Nevertheless, Txnrd1-deficient livers did not accumulate oxidized thioredoxin, oxidized glutathione, carbonylated proteins, or peroxidated lipids (ESS, GFM, and EES, unpublished; Fig. S3), suggesting that the hepatocytes were not oxidatively stressed under standard care conditions. Also, survival and histology suggested Txnrd1-deficient hepatocytes were not severely compromised (Fig. 1). Two general survival/response strategies might be envisaged to account for this: a general “Dauer-like strategy”, where metabolic pathways and their associated oxidative stress are repressed to a minimal state; or a focused “compensatory strategy”, where alternative systems are activated to remunerate deficiencies stemming from Txnrd1 ablation. To distinguish these possibilities, we compared the transcriptomes of *txnrd1^−/−^* and *txnrd1^cond/+^* livers (Fig. 2A). The general response to the mutation was globally subtle. Of 2.6×10^4^ probe sets that hybridized to liver RNAs, signals for only 0.3% (84 probe sets representing 68 different mRNAs) reliably differed by 1.5-fold or more (Fig. 2A; Table S1). Thus, global hepatic activities were generally unaffected by the mutation. Moreover this limited transcriptome response was largely (82%) inductive (Fig. 2A, Table S1). This strongly suggested the response to hepatocytic Txnrd1 deficiency was focused, compensatory, and physiologically effective.

Few differentially abundant mRNAs encoded proteins with activities akin to those of Txnrd1. Thus, mRNAs for Txnrd2 (Fig. 2B), Txnrd3, Txn1, Txn2, GSR, or Grxs were not induced (Table S1 and not shown). Rather, most induced mRNAs encoded xenobiotic/drug metabolism phase I, II, and III enzymes [23], [24]. Hepatic xenobiotic/drug metabolism enzymes constitute a system that is induced in response to exogenous challenges. Phase I enzymes are typically oxidases, phase II enzymes are transferases or other modifying enzymes, and phase III enzymes are exporters [23]–[25]. In Txnrd1-deficient livers, mRNAs encoding the phase I enzymes cytochrome P450 oxidase 2a4 (Cyp2a4), Cyp2b10, Cyp2b13, Cyp8b1, aldehyde oxidase-1 (Aox1), and NADPH-quinine oxidase-1 (Nqo1) were induced (Figs. 2A, B; Table S1). Induced mRNAs for phase II enzymes included those encoding glutathione-S-transferase α1 (GSTα1), GSTα2, GSTα4, GSTμ1, GSTμ3, GSTμ4, microsomal GST3 (MGST3), and UDP glucoronosyltransferase family 2B35 (Ugt2b35). Induced mRNAs for phase III exporters included those encoding the multi-drug resistance-associated ATP-binding cassette sub-family C members 3 and 4 (Abcc3 and Abcc4) (Figs 2A, 2B; Table S1).

For selected mRNAs whose levels were increased in the Txnrd1-deficient livers, we investigated whether this response was transcriptional or post-transcriptional. Previous studies established a correlation between changes in levels of nuclear pre-mRNAs and changes in transcription rates [19], [26]. Nuclei were isolated from *txnrd1^−/−^* and *txnrd1^cond/+^* livers. RT-PCR analyses for representative mRNAs from the array, including those encoding Cbr3, Nqo1, Abcc3, Abcc4, Aox1, GSTμ3, and sulfiredoxin-1 (Srx1), showed higher levels in the *txnrd1^−/−^* nuclei (Fig. 2C). This suggested the transcriptome response was transcriptionally regulated.

**Figure 3:**
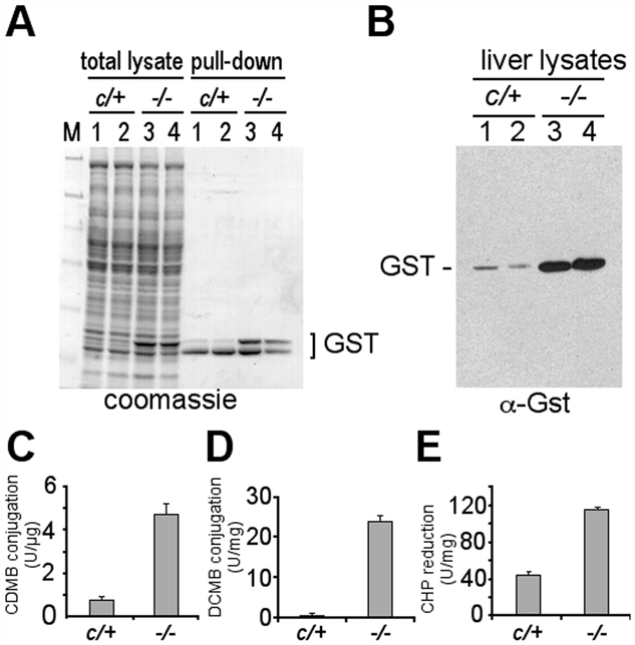
Disruption of hepatocytic Txnrd1 induces α- and μ-class GSTs. (A) Accumulation of GST proteins. Left four lanes, a fraction of total lysates from livers of the indicated genotypes. Right, native GST proteins were pulled-down from the same lysates with glutathione beads. Samples were separated by SDS-polyacrylamide (10%) gel electrophoresis and were stained with coomassie. M, molecular size markers. (B) Anti-GST western blot of proteins separated on SDS-polyacrylamide (15%) gel. (C–E) GST-activity assays using as substrates: (C) 1-chloro-2,4-dinitrobenzene (CDNB) to measure total glutathione-transferase activity; (D) 3,4-dichloronitrobenzene (DCNB) to preferentially measure μ-class GST glutathione transferase activity; and (E) cumene hydroperoxide (CHP) to preferentially measure α-class GST hydroperoxidase activity. Duplicate analyses were performed on two animals of each genotype; graphs show average and S.E.M.

Based on the raw hybridization signals on the arrays, the most highly abundant group of mRNAs in the *txnrd1^−/−^* response were those encoding GSTs (Table S1). GSTs are phase II enzymes that conjugate GSH to electrophilic compounds, thus reducing cellular toxicity. The GST gene super-family contains five families, of which α, μ, and π are most abundant in mammalian cells, and some single-gene members, including MGST3 [27]. Gels of total proteins from control and Txnrd1-null livers showed strong enrichment of a band near 26 kD (Fig. 3A). GSH pull-down analyses confirmed that the enriched band contained GSTs of the size of α- and μ-class, whereas the slightly smaller π-class GST proteins were not enriched in mutants (Fig. 3A) and their cognate mRNAs were not more abundant in the transcriptome data (Table S1). Western blots further verified enrichment of GSTα- or μ-class proteins in *txnrd1^−/−^* liver (Fig. 3B). As compared to control livers, overall GST-transferase activity was 5-fold increased (Fig. 3C); GSTμ-class transferase activity was 100-fold increased (Fig. 3D); and GSTα-class organic peroxidase activity was 2.8-fold increased in *txnrd1^−/−^* livers (Fig. 3E). These results strongly suggested that, although the mice were not challenged by exogenous toxins, the *txnrd1* deficient livers had activated their xenobiotic defense systems.

### Participation of Nrf2 In Response to Txnrd1 Deficiency

**Figure 4:**
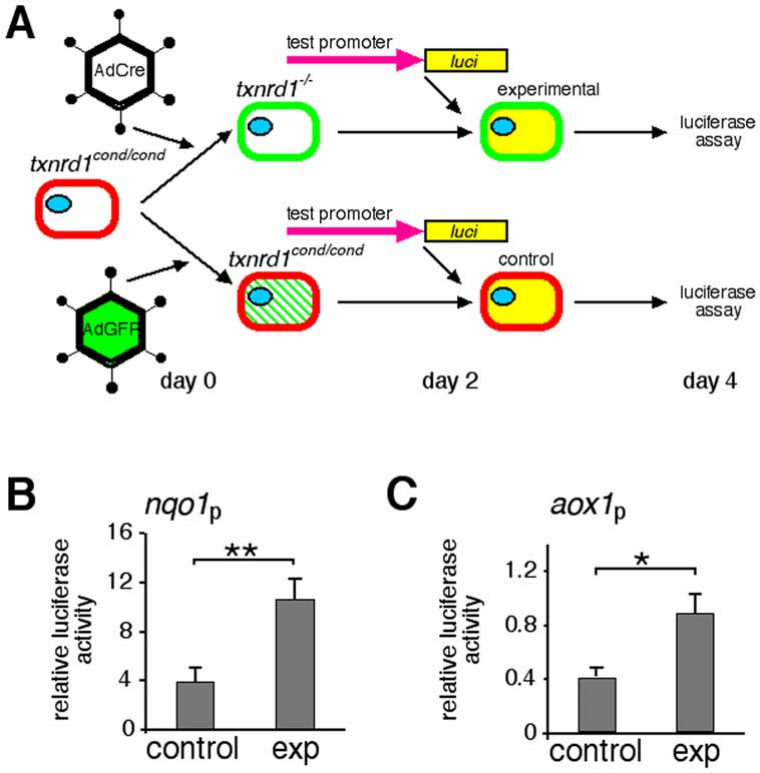
Disruption of Txnrd1 in primary fibroblasts activates the *nqo1* and *aox1* gene proximal promoters. (A) Experimental schematic. MEF cultures initiated from *txnrd1^cond/cond^*;*ROSA^mT-mG/+^* fetuses were transduced with AdCre (experimental) or AdGFP (control). Two days later, each culture was transfected with either *nqo1-luci* or *aox1-luci*. Two days after that, luciferase activities were measured. Red or green membrane fluorescence indicates status of *ROSA^mT-mG^* allele and, by inference, the status of the *txnrd1* allele. The *txnrd1* allelic status is indicated above each cell. Green slashes in cytoplasm denote transient cytoplasmic expression of AdGFP. Yellow cytoplasm denotes luciferase expression. (B) Activity of the *nqo1* promoter (*nqo1*p). Values are in arbitrary units and represent the average of five biological replicates (i.e., MEF cultures from five different fetuses). Data were normalized to expression of *CMV-luci* to control for differences in transfection efficiency that might result from disruption of *txnrd1* or conversion of *ROSA^mT-mG^*. (C) Activity of *aox1* promoter. Like panel (B), but using *aox1-luci* on three biological replicates. Error bars, S.E.M. Asterisks: *, *p*≤0.05; **, *p*≤0.01.

To investigate mechanisms underlying the transcriptome response, a Txnrd1-deficient cell culture system was developed (Figs. 4A, S1). Transduction of *txnrd1^cond/cond^*;*ROSA^mT-mG/+^* MEF cultures with AdCre caused conversion to *txnrd1^−/−^* in >90% of the cells in each culture (Fig. S1). Proximal promoter sequences from the *nqo1* and *aox1* genes (−985 to +116 and −886 to +4 from cap sites, respectively) were fused to a luciferase cassette to generate *nqo1-luci* and *aox1-luci* reporter plasmids. *txnrd1^cond/cond^*;*ROSA^mT-mG/+^* MEF cultures were split and portions were transduced with either AdCre (experimental) or AdGFP (control). After allowing time for pre-formed Txnrd1 protein to decay, cells were transfected with the reporter plasmids. A CMV promoter-driven plasmid was used to control for differences in transfection efficiencies that might result from Txnrd1 disruption and luciferase activities were measured two days later (Fig. 4B). Relative activities of the *aox1-* or *nqo1*-promoters were two- to three-fold higher in the Txnrd1-deficient cultures than in the control cultures (Fig. 4B), which indicated that disruption of Txnrd1 in MEFs resulted in transcriptional induction of the *aox1* and *nqo1* proximal promoters. This directed our focus to these sequences.

Activation of the *nqo1* gene is a hallmark of oxidative or chemical stress in mammalian cells [28]. In this response, *nqo1* is induced by transcription factor Nrf2 binding to an antioxidant response element (ARE) located in the proximal promoter [29]. The promoter of *aox1* is not well characterized; however, we identified two putative AREs located in this region. Also, more than half of the xenobiotic/drug metabolism mRNAs that were induced in the *txnrd1^−/−^* liver transcriptome (Fig. 2, Table S1) reportedly respond to the Nrf2 pathway [7], [8], [30]. Several other mRNAs that were induced but are not classified as xenobiotic/drug metabolism mRNAs, for example those encoding Gpx2 and Srx1, also respond to Nrf2 [7], [31]. Thus, we hypothesized that Nrf2 participated in the response to Txnrd1 deletion.

**Figure 5:**
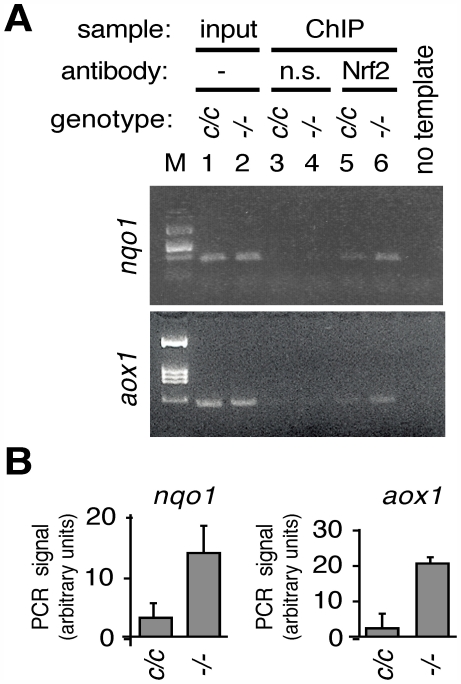
*In Vivo* Assembly of Nrf2 on the *nqo1* and *aox1* Gene Promoters Following Disruption of Txnrd1. (A) MEF cultures from *txnr1^cond/cond^* fetuses were transduced with AdGFP (control, genotype “c/c”) or AdCre (experimental, genotype “−/−”) as in Fig. 4. Chromatin immunoprecipitations were performed using equal amounts of input chromatin for each reaction and affinity purified rabbit-anti-mouse Nrf2 (Nrf2) antibody or a negative control (n.s., non-specific antibody). Relative amounts of the ARE containing regions of the *nqo1* promoter (upper panel) or *aox1* promoter (lower panel) in immunoprecipitates were detected by PCR. Panels show representative PCR data for *nqo1* (upper panel) or *aox1* (lower panel) gene regulatory regions. Input is 5% of the amount of chromatin that was used in each immunoprecipitation. M, molecular size markers. (B) Specific occupancy of Nrf2 on each promoter. Values above background (i.e., Nrf2 antibody-immunoprecipitated minus non-specific antibody-immunoprecipitated) were calculated and presented for MEFs of each genotype. Duplicate analyses were performed on two biological replicates for each gene; graphs show mean and S.E.M.

Because Txnrd1-deficient MEFs showed activation of the ARE-containing *nqo1* and *aox1* proximal promoters (Fig. 4B), these cells were expected to provide a suitable system for measuring whether Nrf2 was directly participating in the response to Txnrd1 disruption. Experimental and control MEF cultures were treated with formaldehyde to cross-link *in vivo* DNA-protein complexes and chromatin immunoprecipitation was performed using anti-Nrf2 antiserum. Occupancy of Nrf2 on the *nqo1* and *aox1* gene regulatory regions was 5-fold and 2.5-fold higher, respectively, in experimental as compared to control cells (Fig. 5B). This indicated that depletion of Txnrd1 resulted in increased *in vivo* occupancy of Nrf2 protein on the AREs of the *nqo1* and *aox1* genes.

**Figure 6:**
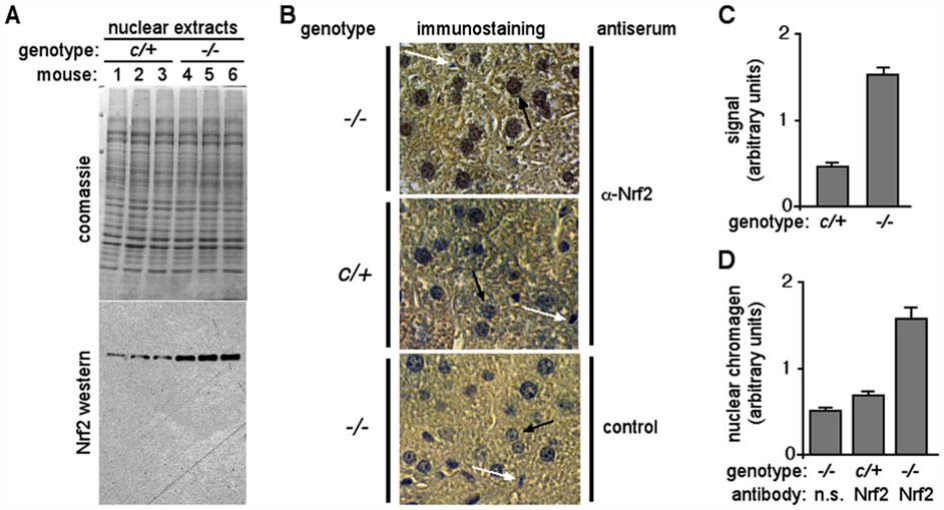
Nuclear enrichment of Nrf2 protein in Txnrd1-deficient hepatocytes. (A) Western blot of Nrf2 protein in nuclear extracts from purified liver nuclei. Livers from three *txnrd1^cond/+^*;*albCre^0^* (*c/+*) and three *txnrd1^cond/−^*;*albCre^1^* (*−/−*) adult female mice were harvested and homogenized in high-viscosity buffer, and nuclei were purified by sedimentation through sucrose/glycerol cushions. Nuclear protein extracts were separated on SDS-polyacrylamide gels and visualized on western blots using affinity purified rabbit-anti-mouse Nrf2 antibody. Top, coomassie-stained gel; bottom, Nrf2 western blot. (B) Immunohistochemistry of Nrf2 in liver. Paraffin sections of livers of the genotypes described for panel (A) were stained using either affinity purified rabbit-anti-Nrf2 antiserum or no primary antibody (control) followed by anti-rabbit horseradish peroxidase conjugate, as indicated. Chromagen development yields a brown color; sections were counter-stained with hematoxylin (blue). The increased brown staining of hepatocytic nuclei (large nuclei, black arrows) in upper panel as opposed to the more blue color of these nuclei in lower two control panels indicated that Nrf2 was more abundant in *txnrd1^−/−^* nuclei. White arrows denote small nuclei, likely of endothelial origin, that did not stain for Nrf2. Scale bars 25 µm. (C) Nrf2-specific band intensities from nuclear extract western blots. Band intensities above background were determined by densitometry of autoradiography films from chemiluminescence western blots. Liver lysates from three animals of each genotype were analyzed; graph represents average and S.E.M. for each genotype. (D) Nrf2-specific nuclear staining by immunohistochemistry. Hematoxylin counter-stains nuclei blue; the brown chromagen from nuclear Nrf2 immunostaining overshadows the blue with brown. To provide a measure of staining, arbitrary hepatocyte nuclei from each genotype were evaluated using Photoshop software for yellow and blue color values. The ratio of yellow:blue was used as a representation of brown chromagen masking of blue hematoxylin staining. Ten hepatocyte nuclei were analyzed from each genotype; graph shows averages and S.E.M.

The Nrf2 pathway typically provides a rapid response to transient challenges [8]. In normal cells, Nrf2 is regulated by a post-translational mechanism that restricts nuclear accumulation of the protein and provides cells with a rapid Nrf2-driven transcriptional response to chemical or oxidative stresses (see Introduction). Based on this mechanism, we reasoned that if Nrf2 were participating in the transcriptome response to Txnrd1 disruption, the protein should preferentially accumulate in *txnrd1^−/−^* liver nuclei. Western blots showed 3-fold enrichment of Nrf2 protein in *txnrd1^−/−^* as compared to control liver nuclear extracts (Figs. 6A, C), and immunostaining for Nrf2 showed increased nuclear staining in many hepatocytes in *txnrd1^−/−^* as compared to control liver cryosections (Figs. 6B, D). This was consistent with Nrf2 participating in the chronic transcriptome response to Txnrd1 deficiency. The results presented here suggest that chronic ablation of hepatocytic Txnrd1 leads to activation of the Nrf2 pathway and induction of genes encoding cytoprotective xenobiotic/drug metabolism enzymes, which participate in an effective compensatory response.

## Discussion

### Mammalian Responses to Txnrd1-disruption

We previously showed that Txnrd1 is essential for embryogenesis [15]. Lethality of *txnrd1^−/−^* embryos was associated with a failure to form mesoderm. Endoderm- and ectoderm-derived cell types differentiated and proliferated, yet in the absence of mesoderm, the embryos were disorganized and did not gastrulate. Single-embryo transcriptome analyses showed induction of some of the same mRNAs observed in *txnrd1^−/−^* liver in the current study, including those encoding several GSTs, Srx1, Cbr3, and others [15]. More recently, we have used the conditional-null allele of *txnrd1* [15] in conjunction with the *albCre* transgene [17] to allow embryos to surpass developmental lethality and generate adults in which all hepatocytes, an endoderm-derived cell type [18], lack Txnrd1. These mice survive and exhibit no overt signs of hepatic oxidative stress; redox states of Txn and GSH are normal and neither peroxidated lipids nor carbonylated proteins accumulate (ESS, GFM, and EES, unpublished data; and Fig. S3). Clearly hepatocytes have a strategy by which chronic hepatocytic ablation of Txnrd1 does not lead to oxidative stress.

The analyses presented here indicated the transcriptome response to chronic Txnrd1 disruption was subtle, involving only ∼0.3% of the mRNAs expressed in liver, and largely inductive (Fig. 2, Table S1). Indeed, only four mRNAs were more than 2-fold less abundant than in controls (Table S1). This suggests survival involves activation of an effective compensatory program.

Studies on bacteria and yeast show that Txnrd-dependent and GSR-dependent pathways can often compensate each other [32]–[34]. More recent studies in plants suggested this might be a general property of all life forms [35]. However, disruption of the *txnrd1* gene in either embryos [15] or adult liver (this study) had no effect on GSR mRNA levels nor on levels of mRNAs encoding Txnrd2, Txnrd3, or Txns (ESS, GFM, and EES, unpublished data). Combined Txnrd + GSR reductase activity levels were two-fold lower in Txnrd1-deficient livers than in control livers (Fig. 1D), confirming that the loss of Txnrd1 enzyme activity was not compensated by posttranscriptional induction of other Txnrds or GSR. Also, unlike Txnrd1-deficient yeast, in which the most dramatically induced mRNAs encode Prxs [36], Prx mRNA levels were not affected in Txnrd1-deficient embryos or livers (Fig. 2B; Table S1) [15](ESS, GFM, and EES, unpublished data). Our studies suggest that something in the evolutionary pathway leading to mammals spurred a departure from universal mechanisms of coordinating reductase systems. Interestingly in this regard, mammalian Txnrd proteins are non-homologous to bacterial or yeast Txnrds, and instead, evolved independently from GSR [37]. One intriguing possibility suggested by our studies might be that, by disabling the ability of GSR-dependent systems to compensate for some aspects of a Txnrd1 deficiency, animals might have been able to assimilate Txnrd1 into stress-response pathways as a component of a redox sensitive trigger (see below).

### Nrf2 Pathway Response to Txnrd1 Deficiency

In the current study, we show that the transcriptome response to chronic hepatocytic Txnrd1 ablation is dominated by induction of mRNAs encoding xenobiotic/drug metabolism enzymes (Table S1). Many of these can respond to the Nrf2 pathway and Nrf2 protein preferentially accumulated in *txnrd1^−/−^* as compared to control liver nuclei (Fig. 6B). Disruption of Txnrd1 in primary fibroblasts resulted in activation of transfected *nqo1* and *aox1* promoters (Fig. 4B) and *in vivo* engagement of endogenous Nrf2 on AREs within endogenous *nqo1* and *aox1* promoters (Fig. 5A). Many of the mRNAs induced in the *txnrd1^−/−^* embryo transcriptome [15] were also Nrf2-response genes. Thus, the response to Txnrd1 deficiency in all mammalian cells may involve activation of the Nrf2 pathway.

The architecture of the Nrf2 pathway allows rapid responses to transient stresses. Nrf2 protein is post-translationally regulated by mechanisms that restrict protein stability and activity [10]. Oxidative stress or electrophilic xenobiotics trigger a reversal of this mechanism resulting in nuclear accumulation of Nrf2. Mice lacking Nrf2 are viable and generally healthy, but they have an impaired ability to respond to oxidative or xenobiotic challenges [8], [12], [31]. It is known that Nrf2 can activate *txnrd1* to help combat transient oxidative challenges [22]; however, it was unexpected that the Nrf2 pathway would respond to Txnrd1 disruption. Instead, we expected that another constitutive electron supplier, like GSR, would compensate for this chronic deficiency. Also, Txnrd1-deficient livers show no evidence of oxidative stress (ESS, GFM, and EES, unpublished data; Fig. S3). This indicates the response pathways revealed in the Txnrd1-deficient liver transcriptome, including induction of mRNAs encoding GSTs, Gpx2, and Srx1 (Table S1), are likely effective at detoxifying ROS.

Importantly, in the absence of measurable oxidative stress, it is unclear what is activating Nrf2. One intriguing possibility is that the Txnrd1/Txn1 system, itself, serves as a part of the Nrf2 stress-response trigger. Thus, active Txnrd1/Txn would maintain the pathway in a repressed state. Challenges that transiently impair Txnrd1/Txn, for example by inducing accumulation of ROS and oxidizing critical sulfhydryls, would de-repress the pathway. Ablation of Txnrd1 in our mice would thus mimic oxidative or xenobiotic challenge and induce a constitutively “on” Nrf2 pathway in the absence of any challenge. Further studies will be required to test this model.

### Mammalian Selenoproteins

Txnrd1 is a selenoprotein. All known selenoproteins are oxidoreductases in which a selenocysteine residue in the carboxyl terminus plays a role in enzyme catalysis at the active site of the protein [38]. Indeed, all orthologues of selenoproteins in all life-forms are predicted oxidoreductases, even though many of these proteins (including, for example, *Drosophila* Txnrds) are not, themselves, selenoproteins [39]. In mice, there are only 24 known selenoproteins. This list includes all three Txnrds, all eight Gpxs, both iodothironine deiodinases, methionine sulfoxide reductase A, and other less well characterized proteins [40].

Disruption of the selenocysteine tRNA locus results in concerted inactivation of all selenoproteins (“selenoprotein-null”), including Txnrd1 [41]. *albCre*-dependent disruption of this locus in hepatocytes results in animals that survive for only 1- to 3-months, after which they succumb to systemic failure associated with hepatocytic and adipocytic necrosis [42]. Prior to death, selenoprotein-null livers exhibit activation of the Nrf2 pathway [43], [44]. However, in these studies, it has been unclear what roles each of the 24 mouse selenoproteins play in the phenotype and what effects might be downstream consequences of necrotic cell death or the deterioration of animal health. Comparisons to the current study will help resolve a part of this uncertainty. We show that disruption of Txnrd1, alone, is sufficient to induce Nrf2 and activate expression of many xenobiotic/drug metabolism genes, as seen in the selenoprotein-deficient mice, but not to cause hepatocytic necrosis or systemic failure. The respective roles of Txnrd2, Gpxs, and other selenoproteins in the exacerbated phenotype of mice bearing selenoprotein-null hepatocytes remain to be determined.

## Materials and Methods

### Mice

All mice were kept under specialized care conditions with a 14 h∶10 h light:dark cycle. Mice bearing the *txnrd1^−^* and *txnrd1^cond^* alleles have been reported previously [15]. Mice bearing the *(ROSA)26Sor^tm4(ACTB-tdTomato,-EGFP)Luo^* [21] (here nick-named “*ROSA^mT-mG^*”) allele, the *(ROSA)26Sor^tm1(cre/Esr1)Nat^* [45] (here nick-named “*ROSA^CreER^*”) allele, and Tg(Alb-cre)21Mgn [17] (here nick-named “*albCre*”) mice were purchased from Jackson Labs (stocks 007576, 004847, and 003574, respectively). Genotypes were determined molecularly for all animals by PCR on genomic DNA using primers indicated in Table S2. All animal protocols were approved by the Montana State University Institutional Animal Care and Use Committee. All renewable resources developed for this study are available for free unrestricted non-profit research use upon request unless specifically restricted by another party.

### Cells and Culture Conditions

Mouse embryo fibroblast cultures (MEFs) were established from E12.5 mouse fetuses as described previously [46]. Cultures were maintained in Dulbecco's minimum essential medium (DMEM) supplemented with 10% fetal bovine serum and 1× penicillin/streptomycin (Mediatech). Experiments were performed within the first five passages. For transductions, cells were lifted off dishes with Accutase (Innovative Cell Technologies), resuspended in fresh medium, and replication-defective AdCre or AdGFP particles were added at a titration to give ∼90% conversion (Fig. S1).

### Transcriptome Analyses

Liver RNA samples were evaluated on Affymetrix mouse version 430 2.0 microarrays. Because liver gene expression may respond to circadian time, feeding, gender, and age, young male animals were singly housed for ten days and harvested between 2:00 and 2:30 p.m. Liver RNA was purified as described [19]. Biotinyllated cRNAs were prepared using the Ambion MessageAmp II-Biotin Enhanced system as per the manufacturer's protocols and array hybridizations, washes, and analyses followed previously reported methods [15].

Txnrd1 is necessary for circadian gene expression in *Neurospora* [47] and it has been suggested that NAD(P):NAD(P)H ratios, which may be sensitive to Txnrd1 disruption, participate in regulating circadian gene expression in mice [48]–[50]. If *txnrd1^−/−^* hepatocytes in mouse livers were acyclic or cycle-shifted, fixed-timed harvests would not control for circadian defects and could impact transcriptome data. Therefore, experimental or control mice as above were harvested at 4 h intervals. Levels of mRNA encoding the D site-binding protein, DBP, an mRNA with a strong circadian cycle [51], and β-actin (control), were assessed by RT-PCR. No measurable effect on the expression of DBP mRNA resulted from *albCre*-driven disruption of Txnrd1 (Fig. S4), which verified that the diurnal gene expression cycle was not affected under these conditions.

Microarray data were analyzed using GeneSpring software (v7.3, Agilent). Probe-sets with an average raw value of ≥50 units across all eight arrays were considered above background and were used for further analysis. Of the 45,101 probe-sets on the arrays, 25,565 met this cut-off. Hybridization signals for each probe-set were considered significantly different between experimental and control samples if they differed by 1.5-fold and had a *p*-value≤0.05 between replicates.

RT-PCR reactions used oligo(dT)-primed cDNAs generated from total RNA preparations with the exception of nuclear pre-mRNA RT-PCR analyses (see below). General reaction conditions were described previously [46]. Primers are listed in Table S2.

### Production of Anti-Nrf2 Antibody

Because commercial antibody raised against Nrf2 oligopeptide antigen proved to be of inadequate quality for these studies (data not shown), we generated our own affinity purified rabbit-anti-mouse Nrf2 antibody. A cDNA fragment corresponding to positions 237 through 2093 of the mouse Nrf2 mRNA (GenBank NM_010902), which encodes the entire Nrf2 open reading frame, was amplified by RT-PCR from C57Bl/6J mouse liver RNA, inserted into pET13a (Novagen) and pGEX4T-1 (Amersham Biosciences) expression vectors, and verified by sequencing. His-tagged recombinant Nrf2 protein was affinity purified on a Ni-column, dialyzed, and used to generate antisera in rabbits. Antibody was affinity purified using the recombinant GST-tagged Nrf2.

### Western Blot Analyses

Proteins were separated by SDS-polyacrylamide gel electrophoresis and were transferred onto nitrocellulose membranes. Membranes were blocked in 10% non-fat milk in 1× PBS containing 0.5% Tween-20 and were incubated overnight with primary antibody. Rabbit-anti-Txnrd1 (Santa Cruz Biotechnology, Inc.), rabbit-anti-Nrf2 (this study), and goat-anti-schistosome GST polyclonal antibody (GE Healthcare #27-4577) polyclonal antibodies were used at 1∶300, 200ng/ml, and 1∶500 dilutions, respectively. BLAST (http://blast.ncbi.nlm.nih.gov/Blast.cgi) analysis indicates that Schistosomes have a single GST protein, which has regions of high amino acid identity with mouse α- and μ-class GSTs but only more scattered identity with other mouse GST classes (not shown). Incubations with horseradish peroxidase-conjugated secondary antibody (1∶30,000; Jackson Immunoresearch Laboratories #711-035-152 and Southern Biotech #6160-05 for anti-rabbit and anti-goat conjugates, respectively) were performed for one hour. Signals were detected using the Supersignal West Pico chemiluminescence system (Pierce).

### Isolation of Liver Nuclei and Purification of Chromatin-free Nuclear Protein Extracts or DNA-free Nuclear RNA

Nuclei isolation from the mouse liver was performed as previously described [19]. Briefly, livers were harvested into ice-cold 1× PBS. Tissue was minced and disrupted using a motor-driven Teflon-glass homogenizer under final conditions of 10% wt/vol minced liver, 1.8 M sucrose, 9% glycerol, 10 mM HEPES, pH 7.6, 15 mM KCl, 0.5% non-fat milk, 5 mM DTT, 0.15 mM spermine, 0.5 mM spermidine, 2 mM EDTA, 1× protease inhibitor (Sigma), and 1 mM PMSF. Lysates were layered onto 10 ml cushions of 2 M sucrose, 10% glycerol, 10 mM HEPES pH 7.6, 15 mM KCl, 1 mM DTT, 0.15 mM spermine, 0.5 mM spermidine, 2 mM EDTA, 0.1× protease inhibitor, and 0.1 mM PMSF in SW-28 tubes and sedimented at 24,000 r.p.m. for 1 h at 4°C in a pre-cooled SW-28 rotor. Nuclei were collected and were either used fresh or snap-frozen in liquid nitrogen and stored at −80°C. Chromatin-free nuclear proteins were extracted from fresh or frozen nuclei with a final concentration of 1 M urea, 1 M NaCl, and 1% NP-40 following previously described methods [52]. DNA-free nuclear RNA was isolated by sedimentation through CsCl cushions as described previously [53]. Since nascent pre-mRNA strands are not yet poly-adenylated, cDNAs for nuclear RT-PCR reactions were random-primed.

### Chromatin Immunoprecipitation

ChIP assays were performed as previously described [54], [55]. Each experiment initiated with eight-10 cm dishes of MEFs at 50% confluence. Four dishes were transduced with AdGFP (∼1.5×10^8^ PFU; control) and four with AdCre (∼1.5×10^8^ PFU; experimental), as above. Dishes were cross-linked with 10 ml of buffer containing a final concentration of 0.93% formaldehyde at room temperature for 10 min. Cross-linking was stopped by adjusting to 120 mM glycine, incubating at room temperature for 5 min, and washing twice with 1× PBS. Rinses were aspirated to completion and cells were harvested by scraping in a total of 5 ml (for 4 dishes) of 10 mM Tris, pH 8, 0.5 mM EDTA, 85 mM KCl, 0.5% Triton X-100, 1× protease inhibitors, 1 mM PMSF. Nuclei were pelleted at 1000×g for 10 min at 4°C, resuspended in 1 ml 50 mM Tris, pH 8, 150 mM NaCl, 1% Triton X-100, 0.1% SDS, 0.5% sodium deoxycholate, 1 mM EDTA. Chromatin was sheared to 400–1000 bp average-length fragments by sonication and debris was removed by centrifugation at 13,000 r.p.m., 10 min, in a microfuge. DNA was purified from a portion of the supernatent by extraction with phenol/chloroform and precipitation with ethanol, and the DNA concentration and size were verified by spectrophotometry and electrophoresis, respectively. An equal amount of input chromatin (50 µg) was used in each immunoprecipitation; the “input” lanes on gels contained 5% as much chromatin (2.5 µg). To reduce non-specific background, chromatin samples were pre-adsorbed with 20 µl of a 50% slurry of protein-A/G agarose beads (Calbiochem #IP05) that had been pre-blocked with sheared salmon sperm DNA and washed extensively with immunoprecipitation buffer. Immunoprecipitations used 25 µl affinity purified anti-Nrf2 antibody or 25 µl of rabbit-anti-HA as a matched control, and 40 µl of the blocked protein-A/G agarose bead slurry. Washes were as described previously [55]. After reversing cross-links and purifying DNA, the abundance of sequences of interest in each immunoprecipitate was analyzed by PCR with primers for the regulatory regions of the indicated genes (Table S2).

### Analyses of Enzyme Activities and GST Pull-down Assays

Unless otherwise indicated, all specialized reagents and substrates for enzyme assays were from Sigma. Mouse livers were homogenized by pulse sonication in ice-cold 1× PBS, pH 7.4, containing 1 mM PMSF and 1× protease inhibitor. Proteins were extracted by addition of Triton X-100 to a final concentration of 1% followed by incubation on ice for 30 min. Debris was removed by centrifugation. Combined reductase activities were determined using 5,5′-dithiobis(2-nitrobenzoic acid) (DTNB) as described previously [56]. This substrate is reduced by all Txnrds and GSR [56]. The value of this assay is that it indicates whether there is a change in the overall reductase capacity of the entire class of enzymes, which would not happen if another reductase of this group simply compensated for loss of Txnrd1. Enzymatic activities toward 1-chloro-2,4-dinitrobenzene (CDNB) and 1,2 dichloro-4-nitrobenzene (DCNB) were measured in 100 mM phosphate buffer, pH 6.5, 1 mM EDTA, 2 mM GSH, 2 mM CDNB or 2 mM DCNB [57]. Conjugation was monitored in a spectrophotometer at 340 nm or 345 nm, respectively. Cumene hydroperoxide (CHP) reduction activity was measured in the coupled GSR catalyzed reaction containing 100 mM phosphate buffer, pH 6.5, 1 mM EDTA, 0.5 mM NADPH, 2 mM GSH, 2.5 mM CHP and 1.5 U/ml yeast GSR. Oxidation of NADPH was detected at 340 nm [57].

For pull-down assays, Triton X-100-solubilized liver extracts were incubated with glutathione-agarose beads (Sigma G-4510). Beads were washed 3 times with ice-cold 1× PBS, pH 7.4 containing 0.5% Triton X-100 and 0.1 mM PMSF. Bound proteins (i.e., GSTs) were eluted by boiling 10 min in loading buffer were separated on SDS-polyacrylamide (10%) gels.

### Luciferase Reporter Assay

The proximal promoter sequences of the *aox1* and *nqo1* genes amplified from C57Bl/6J genomic DNA using the primers indicated in Table S2 were inserted into plasmid pGL3 (Promega). For *nqo1*, sequences spanned from −985 to +116 from the cap site; for *aox1,* sequences spanned from −886 to +4. Resultant plasmids “*nqo1-luci*” and “*aox1-luci*” were sequenced and were used in transfections of MEFs prepared as described above. Control transfections were performed with pGL3 vector containing 499 bp of the CMV promoter, *CMV-luc*. Cells were transduced with 1.5×10^8^ PFU of either AdGFP or AdCre. Two days post-transduction, cultures were seeded into 12-well dishes and transfected in triplicate with 1.6 µg of luciferase reporter plasmid using NovaFector transfection reagent (Venn Nova, Inc.). Luciferase activity was measured two days post-transfection.

### Immunohistochemistry

Livers were fixed in formalin and embedded in paraffin. Deparaffinized sections (5 µm) were either stained with H&E or used for immunohistochemistry. For antigen retrieval, sections were heated in 100 mM sodium citrate, 90°C, for 10 min followed by gradual cooling to room temperature. Endogenous peroxidases were inactivated with 0.3% H_2_O_2_ for 10 min at room temperature and sections were blocked with 5% BSA in 1× PBST for one hour. Incubation with affinity purified anti-Nrf2 antibody was performed overnight at room temperature followed by standard washes and one-hour incubation with goat HRP-conjugated anti-rabbit IgG (Bio-Rad). Following washes, sections were developed using diaminobenzamidine at pH 7.0 and counter-stained with hematoxylin.

## Supporting Information

Figure S1 Correlation between *ROSA^mT-mG^* allelic conversion and *txnrd1^cond^* conversion (A) Efficiency of *ROSA^mT-mG^* conversion in primary mouse embryo fibroblasts (MEFs). Primary fibroblast cultures were initiated from E12.5 *txnrd1^cond/cond^*;*ROSA^mT-mG/+^* fetuses. Cultures were transduced with 1 x 108 PFU AdCre vector and were photographed one- and three-days later (1d and 3d, respectively). At 1d, most MEFs exhibited both red and green fluorescence; by 3d, MEFs were either red (non-converted) or green (converted and the red-fluorescent tdTomato protein had decayed). Cell counts revealed that ∼90% of the MEFs converted to green fluorescence. (B) Efficiency of txnrd1cond conversion in MEFs. DNA was isolated from the cultures at 1, 3, and 5 days after transduction and relative levels of the txnrd1cond allele (top panel), the txnrd1- allele (middle panel), and an arbitrary genomic control allele (the aox1 gene promoter; bottom panel) were measured by PCR. As a control with equimolar representation of both of these *txnrd1* alleles, DNA from a *txnrd1^cond/−^* mouse was used (second lane from right). n.t., no template control.
(0.24 MB JPG)

Figure S2 Extent and persistence of *txnrd1* ^−/−^ hepatocytes in mice (A) All hepatocytes in aged txnrd1cond/-;ROSA^mT−mG/+^;*albCre* ^1^ mice exhibit allelic conversion. Panels show red, green, and merged red + green + blue fluorescence of a cryosection of liver from a 280 day−old mouse. Capillaries are lined with red-membrane *txnrd1^cond/−^* endothelium (white arrows) because these cells do not express albCre. Other non-hepatocytic cell types, such as Kupffer cells, would also not express *albCre* and would be *txnrd1^cond/−^* and red. All hepatocytes have green membranes. Scale bars 50 μm. (B) Persistence of *txnrd1^−/−^* cells and/or their progeny. *txnrd1cond/^cond^*;*ROSA^mT-mG/mT-mG^* dams were mated by *txnrd1^cond/cond^*;*ROSA^CreER/CreER^* sires and at E14.5, dams received a single I.P. injection of 500 μg 4-hydroxy Tamoxifen (4OHT) in vegetable oil. At P22 (∼27 days after 4OHT administration) a pup was sacrificed and liver cryosections were prepared. Figure shows merged-color fluoromicrographs taken at low-, medium-, and high-magnification. Low magnification shows that the liver was mosaic, with ∼50% of cells being green *txnrd1^−/−^* and 50% being red txnrd1cond/cond. Yellow arrows indicate binucleate green *txnrd1^−/−^* hepatocytes. Because allelic conversion would only occur during a finite period following 4OHT administration, all green cells must either be cells, or be descendents of cells, that converted to *txnrd1^−/−^* ∼27 days earlier, indicating that individual *txnrd1^−/−^* cells are long-term viable.
(7.57 MB DOC)

Figure S3 Txnrd1-deficient livers do not accumulate oxidized glutathione or peroxidized lipids Livers were harvested from four *txnrd1^cond/+^*;*albCre* ^0^ (control) and four *txnrd1^cond/−^*;*albCre^1^* (experimental) adult male mice and lysates were prepared as indicated in Materials and Methods. (A) Levels of total and oxidized glutathione were measured in acid-precipitated liver cytosols using a coupled glutathione reductases assay [57]. Liver was homogenized in the presence (bars labeled "GSSG") or absence (bars labeled "total") of 0.1M N-ethyl maleimide (NEM), which blocked all reduced glutathione. Clarified cytosols were precipitated with 5% sulfosalicylic acid, which denatured all endogenous reductases and oxidized all reduced glutathione in the untreated sample. The precipitate was resuspended and levels of oxidized glutathione were measured in a coupled reaction using 0.15 mM DTNB, 0.2mM NADPH, and 1 U/ml yeast glutathione reductases (Sigma). Change in absorbance at 405 nm was monitored and glutathione content was calculated using DTNB molar extinction coefficient of 14.15 x 105 M-1 cm-1 [58], since the molar ratio of converted DTNB to GSSG in these coupled reaction is 1:1. (B) Lipid peroxidation was assayed by measuring thiobarbituric acid-reactive substance (TBARS) as previously described [59]. Mouse liver was homogenized in the presence of 5mM butylated hydroxytoluene (BHT), and then mixed with 2 volumes of TBA solution (15% trichloroacetic acid, 0.2 M HCl, 0.37% thiobarbituric acid, 0.03% BHT). The mixture was boiled for 1 h and clarified by centrifugation. Absorbance of clarified supernatant was recorded at 535nm. The amount of TBARS was calculated based on the extinction coefficient of malondialdehyde at a wavelength of 535 nm (1.56 x 105 M-1 cm-1) [59]. Graphs in both panels show average and standard deviation for assays on four experimental and four control livers.
(0.08 MB JPG)

Figure S4 Disruption of Hepatocytic Txnrd1 Does Not Affect Diurnal Expression of DBP mRNA, a Circadian Regulated Transcript, in Liver or Kidney Young male mice of the indicated genotypes were singly housed for 10 days under a normal light cycle. Beginning on the 10th day, a single experimental and a single control animal were sacrificed at 4 h intervals for a complete 24 h cycle. Liver and kidney were harvested and RNA was prepared. Olio(dT)-primed RT-PCRs for the mRNAs indicated at left were performed on each sample. Primer sequences for each RT-PCR reaction are in Table S2. Circadian time of harvest (24 h clock) is indicated above each experimental lane and the light cycle is indicated at bottom. n.t., no template control. Results showed that the cyclic expression of DBP mRNA was not measurably affected by hepatocytic disruption of Txnrd1 (peak between 12:00 and 16:00) in either liver or kidney.
(0.16 MB JPG)

Table S1 a Transcriptome data from the livers of four animals of each genotype is presented. For inclusion, a probe-set had to show > 1.5-fold difference in abundance between genotypes, an average hybridization value across all 8 arrays of 50 units, and a p-value of < 0.05. In cases where multiple probe-sets for a single gene existed, only one is shown. All raw data are publicly available in the Geo system at http://www.ncbi.nlm.nih.gov/geo/, accession number GSE16381. b Difference is fold-change, with positive values being higher and negative values being lower in *txnrd1^−/−^* livers. c Average signals for the four biological replicates of the genotype with higher mRNA abundance. For mRNAs with a positive difference value, the *txnrd1^−/−^* average signal is given; for mRNAs with negative difference values, the *txnrd1^cond/+^* average signal is given. d Average signal of three replicates is given e The probe-set used for this entry does not distinguish between Gsta1 and 2.
(0.11 MB DOC)

Table S2 a All primers are designed using mouse sequences; however some have non-mouse 5' extensions. b Genomic primers for genotyping and in ChIP assay. c RT-PCR designed to cDNA sequences. Most but not all span at least one exon/exon junction. d Primer sets used for cloning proximal promoter regions of *aox1* or *nqo1* genes for reporter constructs or for cloning Nrf2 open reading frame (ORF) for recombinant protein expression.
(0.61 MB DOC)
