# Supplementary figures and images for: Cytoprotective Nrf2 Pathway Is Induced In Chronically Txnrd 1-Deficient Hepatocytes
Source: PLoS One. 2009 Jul 7;4(7):e6158. doi: 10.1371/journal.pone.0006158 (PMC2703566; doi:10.1371/journal.pone.0006158)

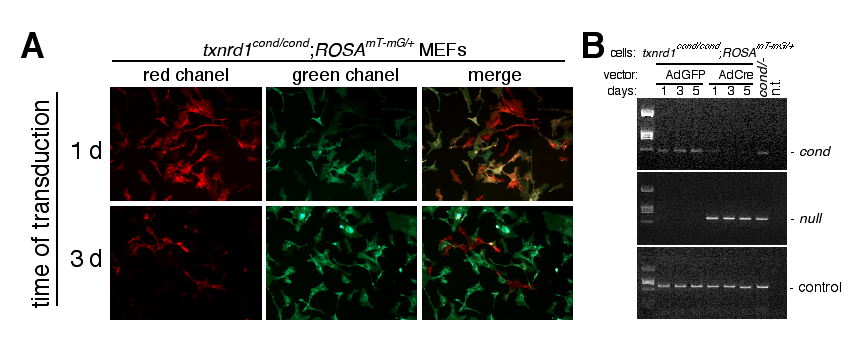

Supplement: Figure S1 — Correlation between ROSAmT-mG allelic conversion and txnrd1cond conversion (A) Efficiency of ROSAmT-mG conversion in primary mouse embryo fibroblasts (MEFs). Primary fibroblast cultures were initiated from E12.5 txnrd1cond/cond;ROSAmT-mG/+ fetuses. Cultures were transduced with 1 x 10^8^ PFU AdCre vector and were photographed one- and three-days later (1d and 3d, respectively). At 1d, most MEFs exhibited both red and green fluorescence; by 3d, MEFs were either red (non-converted) or green (converted and the red-fluorescent tdTomato protein had decayed). Cell counts revealed that ∼90% of the MEFs converted to green fluorescence. (B) Efficiency of txnrd1cond conversion in MEFs. DNA was isolated from the cultures at 1, 3, and 5 days after transduction and relative levels of the txnrd1cond allele (top panel), the txnrd1- allele (middle panel), and an arbitrary genomic control allele (the aox1 gene promoter; bottom panel) were measured by PCR. As a control with equimolar representation of both of these txnrd1 alleles, DNA from a txnrd1cond/− mouse was used (second lane from right). n.t., no template control. (0.24 MB JPG) [file pone.0006158.s001.jpg]

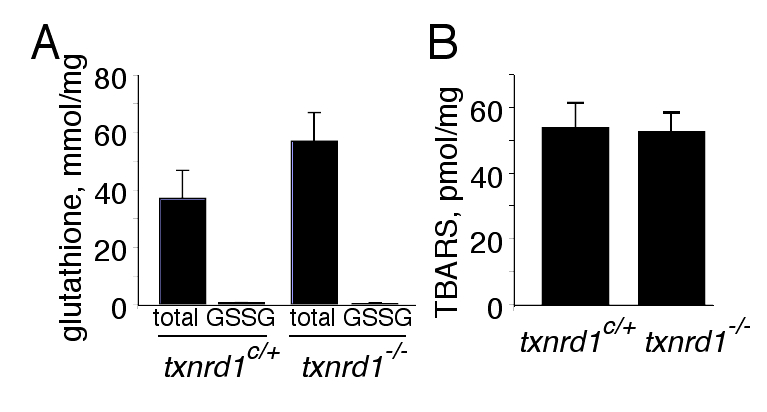

Supplement: Figure S3 — Txnrd1-deficient livers do not accumulate oxidized glutathione or peroxidized lipids Livers were harvested from four txnrd1cond/+;albCre 0 (control) and four txnrd1cond/−;albCre1 (experimental) adult male mice and lysates were prepared as indicated in Materials and Methods. (A) Levels of total and oxidized glutathione were measured in acid-precipitated liver cytosols using a coupled glutathione reductases assay [57]. Liver was homogenized in the presence (bars labeled "GSSG") or absence (bars labeled "total") of 0.1M N-ethyl maleimide (NEM), which blocked all reduced glutathione. Clarified cytosols were precipitated with 5% sulfosalicylic acid, which denatured all endogenous reductases and oxidized all reduced glutathione in the untreated sample. The precipitate was resuspended and levels of oxidized glutathione were measured in a coupled reaction using 0.15 mM DTNB, 0.2mM NADPH, and 1 U/ml yeast glutathione reductases (Sigma). Change in absorbance at 405 nm was monitored and glutathione content was calculated using DTNB molar extinction coefficient of 14.15 x 105 M-1 cm-1 [58], since the molar ratio of converted DTNB to GSSG in these coupled reaction is 1:1. (B) Lipid peroxidation was assayed by measuring thiobarbituric acid-reactive substance (TBARS) as previously described [59]. Mouse liver was homogenized in the presence of 5mM butylated hydroxytoluene (BHT), and then mixed with 2 volumes of TBA solution (15% trichloroacetic acid, 0.2 M HCl, 0.37% thiobarbituric acid, 0.03% BHT). The mixture was boiled for 1 h and clarified by centrifugation. Absorbance of clarified supernatant was recorded at 535nm. The amount of TBARS was calculated based on the extinction coefficient of malondialdehyde at a wavelength of 535 nm (1.56 x 10^5^ M-1 cm-1) [59]. Graphs in both panels show average and standard deviation for assays on four experimental and four control livers. (0.08 MB JPG) [file pone.0006158.s003.jpg]

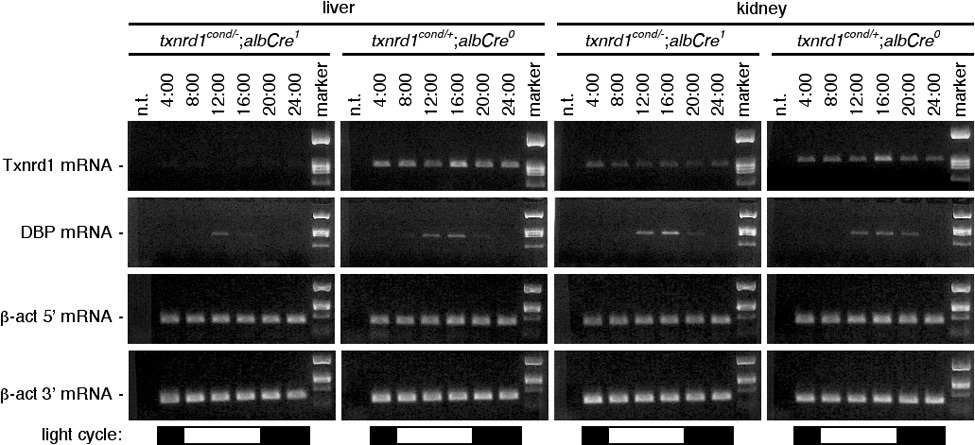

Supplement: Figure S4 — Disruption of Hepatocytic Txnrd1 Does Not Affect Diurnal Expression of DBP mRNA, a Circadian Regulated Transcript, in Liver or Kidney Young male mice of the indicated genotypes were singly housed for 10 days under a normal light cycle. Beginning on the 10th day, a single experimental and a single control animal were sacrificed at 4 h intervals for a complete 24 h cycle. Liver and kidney were harvested and RNA was prepared. Olio(dT)-primed RT-PCRs for the mRNAs indicated at left were performed on each sample. Primer sequences for each RT-PCR reaction are in Table S2. Circadian time of harvest (24 h clock) is indicated above each experimental lane and the light cycle is indicated at bottom. n.t., no template control. Results showed that the cyclic expression of DBP mRNA was not measurably affected by hepatocytic disruption of Txnrd1 (peak between 12:00 and 16:00) in either liver or kidney. (0.16 MB JPG) [file pone.0006158.s004.jpg]
