# Supplementary material for: Cytoprotective Nrf2 Pathway Is Induced In Chronically Txnrd 1-Deficient Hepatocytes
Source: PLoS One. 2009 Jul 7;4(7):e6158. doi: 10.1371/journal.pone.0006158 (PMC2703566; doi:10.1371/journal.pone.0006158)
Supplement: Table S1 — a Transcriptome data from the livers of four animals of each genotype is presented. For inclusion, a probe-set had to show > 1.5-fold difference in abundance between genotypes, an average hybridization value across all 8 arrays of 50 units, and a p-value of < 0.05. In cases where multiple probe-sets for a single gene existed, only one is shown. All raw data are publicly available in the Geo system at http://www.ncbi.nlm.nih.gov/geo/, accession number GSE16381. b Difference is fold-change, with positive values being higher and negative values being lower in txnrd1−/− livers. c Average signals for the four biological replicates of the genotype with higher mRNA abundance. For mRNAs with a positive difference value, the txnrd1−/− average signal is given; for mRNAs with negative difference values, the txnrd1cond/+ average signal is given. d Average signal of three replicates is given e The probe-set used for this entry does not distinguish between Gsta1 and 2. (0.11 MB DOC) [file pone.0006158.s005.doc]

**Table S1.** mRNAs Differentially Expressed in *txnrd1^cond/+^*;*albCre^0^* and *txnrd1^-/-^*;*albCre^1^* Livers^a^

| mRNA | Difference^b^ | Signal^c^ | *p-*value | Description | GenBank |
| --- | --- | --- | --- | --- | --- |
| Cbr3 | 51.7 | 3012 | 0.001 | Carbonyl reductase 3 | AK003232 |
| Gstα1,2^d^ | 29.1 | 9304 | 0.000 | Glutathione S-transferase, α1, 2 | NM_008181 |
| Gstα2 | 14.3 | 7546 | 0.000 | Glutathione S-transferase, α2 | NM_008182 |
| Abcc4 | 13.2 | 998 | 0.000 | ATP-binding cassette, subfamily C, member 4 | BB291885 |
| EST | 10.2 | 956 | 0.013 | Similar to fatty acid desaturase | AV378018 |
| Nqo1 | 6.0 | 986 | 0.000 | NAD(P)H dehydrogenase, quinone 1 | AV158882 |
| Aox1 | 4.6 | 3594 | 0.001 | Aldehyde oxidase 1 | NM_009676 |
| Gstµ3 | 4.5 | 5497 | 0.001 | Glutathione S-transferase,µ3 | J03953 |
| Cyp2b13 | 3.6 | 967 | 0.044 | Cytochrome P450, family 2, subfamily b, polypeptide 13 | NM_007813 |
| Gstµ4 | 3.4 | 3121 | 0.001 | Glutathione S-transferase, µ4 | AF464943 |
| Ugdh | 3.3 | 7076 | 0.001 | UDP-glucose dehydrogenase | NM_009466 |
| Abcc3 | 3.1 | 3297 | 0.000 | ATP-binding cassette, subfamily C, member 3 | AK006128 |
| Gstα4 | 2.8 | 3330 | 0.001 | Glutathione S-transferase, alpha 4 | NM_010357 |
| Sxrn1 | 2.8 | 1021 | 0.010 | Sulfiredoxin 1 | BM210600 |
| Amn | 2.7 | 105 | 0.000 | Amnionless | NM_033603 |
| Cyp2a4 | 2.7 | 17965 | 0.000 | Cytochrome P450, family 2, subfamily a, polypeptide 4 | NM_007812 |
| EST | 2.6 | 1064 | 0.004 | EST; GI:20350159 | BQ174667 |
| Akr1c19 | 2.5 | 3446 | 0.011 | Aldo-keto reductase family 1, member C19 | BG073853 |
| Mgst3 | 2.5 | 1400 | 0.000 | Microsomal glutathione S-transferase 3 | NM_025569 |
| Cyp8b1 | 2.4 | 3906 | 0.015 | Cytochrome P450, family 8, subfamily b, polypeptide 1 | BC010973 |
| Slc48a1 | 2.4 | 1046 | 0.041 | Solute carrier family 48 (heme transporter) member 1 | BC022913 |
| Entpd5 | 2.3 | 3358 | 0.006 | Ectonucleoside triphosphate diphosphohydrolase 5 | NM_007647 |
| EST | 2.3 | 643 | 0.003 | RIKEN cDNA 0610012H03 gene | AK002603 |
| EST | 2.3 | 175 | 0.045 | RIKEN cDNA 0610008F07 gene | BC025862 |
| Accn5 | 2.3 | 188 | 0.018 | Amiloride-sensitive cation channel 5, intestinal | NM_021370 |
| Asns | 2.3 | 161 | 0.049 | Asparagine synthetase | AV212753 |
| Cd36 | 2.3 | 961 | 0.016 | CD36 antigen | BB534670 |
| Cbr1 | 2.2 | 5093 | 0.006 | Carbonyl reductase 1 | NM_007620 |
| Pir | 2.2 | 1036 | 0.003 | Pirin | AK009757 |
| Htatip2 | 2.2 | 2613 | 0.001 | HIV-1 TAT interactive protein 2, homolog (human) | AF061972 |
| Adora1 | 2.2 | 229 | 0.008 | Adenosine A1 receptor | BE630294 |
| Cryl1 | 2.2 | 384 | 0.023 | Crystallin, lamda 1 | NM_030004 |
| EST | 2.2 | 663 | 0.017 | Hypothetical gene supported by BC047216 | AV066667 |
| Ugt2B35 | 2.2 | 8306 | 0.009 | UDP-glucuronosyltransferase 2 family, polypeptide 35 | AA572504 |
| Lrtm1 | 2.1 | 121 | 0.010 | Leucine-rich repeats and transmembrane domains 1 | AV359781 |
| Ppfibp2 | 2.1 | 624 | 0.013 | Protein tyrosine phosphatase, CCLP1 | NM_008905 |
| Ephx1 | 2.1 | 10732 | 0.003 | Epoxide hydrolase 1, microsomal | NM_010145 |
| Gpx2 | 2.1 | 204 | 0.029 | Glutathione peroxidase 2 | NM_030677 |
| Gstµ2 | 2.1 | 3102 | 0.005 | Glutathione S-transferase, µ2 | NM_008183 |
| Gclc | 2.1 | 5315 | 0.018 | Glutamate-cysteine ligase, catalytic subunit | AW825835 |
| Ly6d | 2.1 | 234 | 0.017 | Lymphocyte antigen 6 complex, locus D | NM_010742 |
| Cyp2b10 | 2.0 | 287 | 0.036 | Cytochrome P450, family 2, subfamily b, polypeptide 10 | AF128849 |
| Ldhd | 1.9 | 878 | 0.016 | Lactate dehydrogenase D | AW105779 |
| Ikbkg | 1.9 | 857 | 0.013 | Inhibitor of kappaβ kinase gamma | BB147462 |
| Blvrb | 1.9 | 3081 | 0.003 | Biliverdin reductase B (flavin reductase (NADPH)) | BC027279 |
| Creg1 | 1.9 | 6006 | 0.004 | Cellular repressor of E1A-stimulated genes 1 | BC027426 |
| Agxt2l1 | 1.9 | 3732 | 0.004 | Alanine-glyoxylate aminotransferase 2-like 1 | AK005060 |
| Gstµ1 | 1.9 | 6325 | 0.025 | Glutathione S-transferase, µ1 | J03952 |
| Cdca8 | 1.9 | 75 | 0.011 | Cell division cycle associated 8 | AV307110 |
| Slc9a6 | 1.8 | 331 | 0.042 | Solute carrier family 9 (sodium/hydrogen exchanger), isoform 6 | BB611738 |
| EST | 1.8 | 102 | 0.026 | RIKEN cDNA D630045J12 gene | BB503889 |
| Tdo2 | 1.7 | 4285 | 0.021 | Tryptophan 2,3-dioxygenase | AI595721 |
| Thnsl2 | 1.7 | 1065 | 0.017 | Threonine synthase-like 2 (bacterial) | BC025604 |
| Ddah1 | 1.5 | 2195 | 0.001 | Dimethylarginine dimethylaminohydrolase 1 | BB770857 |
| Acot1 | -1.5 | 492 | 0.045 | Acyl-CoA thioesterase 1 | NM_134188 |
| Acly | -1.5 | 919 | 0.028 | ATP citrate lyase | BI456232 |
| Avpr1a | -1.6 | 530 | 0.031 | Arginine vasopressin receptor 1A | D49729 |
| Fasn | -1.6 | 2535 | 0.046 | Fatty acid synthase | AF127033 |
| Fads2 | -1.8 | 45678 | 0.014 | Fatty acid desaturase 2 | NM_019699 |
| Gck | -1.9 | 1067 | 0.032 | Glucokinase | BC011139 |
| E2f8 | -1.9 | 467 | 0.028 | E2F transcription factor 8 | NM_001013368 |
| EST | -2.2 | 318 | 0.048 | RIKEN cDNA 4432416J03 gene | BB138760 |
| Prtn3 | -2.3 | 106 | 0.030 | Proteinase 3 | U97073 |
| Fabp5 | -2.4 | 3813 | 0.042 | Fatty acid binding protein 5, epidermal | BC002008 |
| Scd1 | -2.7 | 880 | 0.001 | Stearoyl-coenzyme A desaturase 1 | NM_009127 |
