# Supplementary material for: Cytoprotective Nrf2 Pathway Is Induced In Chronically Txnrd 1-Deficient Hepatocytes
Source: PLoS One. 2009 Jul 7;4(7):e6158. doi: 10.1371/journal.pone.0006158 (PMC2703566; doi:10.1371/journal.pone.0006158)
Supplement: Table S2 — a All primers are designed using mouse sequences; however some have non-mouse 5' extensions. b Genomic primers for genotyping and in ChIP assay. c RT-PCR designed to cDNA sequences. Most but not all span at least one exon/exon junction. d Primer sets used for cloning proximal promoter regions of aox1 or nqo1 genes for reporter constructs or for cloning Nrf2 open reading frame (ORF) for recombinant protein expression. (0.61 MB DOC) [file pone.0006158.s006.doc]

**Table S2.** Primer Sequences

| Gene^a^ | Forward sequence | Reverse sequence |
| --- | --- | --- |
| **Genomic PCR**^b^ | | |
| *txnrd1*^cond^ | 5’-TATACTAGTGCTGGTCTTGGATTTTGTCAC-3’ | 5’-CTGCTGAACCTAAATCTACAGCACTG-3’ |
| *txnrd1*^-^ | 5’-CAACAGATTGCATGCTCTTGG-3’ | 5’-CTGCTGAACCTAAATCTACAGCACTG-3’ |
| *aox1* ARE region | 5’-TATAAGCTTGGTCTCATTTGAGACTTGCCAGGT-3’ | 5’-TATGGATCCTAAAGGGAAAACTCTCCTGACA-3’ |
| *nqo1* ARE region | 5’-TATGGATCCGCAGTTTCTAAGAGCAGAACG | 5’-TATGAATTCTCGTGGGACCTGCCTACATAATCA-3’ |
| *alb* proximal promoter | 5’-TATAGATCTTCATTACCTGTACATAAAAGCA-3’ | 5’-TATGGATCCAGAGGCTAGTGGGGTTGATAG-3’ |
| **RT-PCR**^c^ | | |
| Abcc3 | 5’-TATGGATCCTGGTTTTAGACGAGGCCACTG-3’ | 5’-TATGAATTCATTTCCTCATTCCACTGTGAGAC-3’ |
| Abcc4 | 5’-TATGGATCCATCATTGACAGTGACAAGATAATG-3’ | 5’-TATGAATTCAAGACAGACTTTACGATGATGTC-3’ |
| Aox1 | 5’-TATGGATCCATGATGCAGTGAAGGCAGCG-3’ | 5’-TATGAATTCCGAGTCGGCTTCATGTAAAGCAGT-3’ |
| β-Actin-3’ | 5’-GCTGTCTGGTGGTACCACCATGTA-3’ | 5’-ATCTGCTGGAAGGTGGACAGTGAG-3’ |
| β-Actin-5’ | 5’-ACGATATCGCTGCGCTGGTCGTCG-3' | 5’-TGGGGTACTTCAGGGTCAGGATAC-3' |
| Cbr3 | 5’-TATGAATTCAGGTGGTCCGTGTGTCCCTCTGA-3’ | 5’-TATAAGCTTGAATGTCGAAGGGTGTTGGGTCA-3’ |
| GSTµ3 | 5’-TATGGATCCTCCCAAGACCTGTGTTTACTAAG-3’ | 5’-TATGAATTCTAAAGGCTGCATGGGCTTGACTGG-3’ |
| Nqo1 | 5’-TATGGATCCATTCCAGCTGACAACCAGATCA-3’ | 5’-TATGAATTCTAGAAGAGTATTTCCAGCTCGCT-3’ |
| Pxrd1 | 5’-TGGGCAGACCAATCTTCTATCAGTCAC-3’ | 5’-TAGGCAGGTAGATCTTTCAGAGGCCA-3’ |
| Pxrd2 | 5’-TCTGGTGAATAGTGATCCTGCCCTGA-3’ | 5’-CATGTCTATGCACGTTCTGCCCATGT-3’ |
| Pxrd4 | 5’-GACTGACTATCGTGGGAAATACTTGG-3’ | 5’-CACAATGACCTTTATTGAGAAGGTCC-3’ |
| Sxrn1 | 5’-TGCAAACCTAGAGTCCAGGAGGCAAT-3’ | 5’-GAAAAGTTGCAGAGACTAGAGTTCCC-3’ |
| Txnrd1 | 5’-TATACTAGTGCTGGTCTTGGATTTTGTCAC-3’ | 5’-ATAGAATTCCAAGGCGACATAGGATGCAC-3’ |
| Txnrd2 | 5’-TATGGATCCATACTGGACGGCAAACCAGAGCT-3’ | 5’-TATGAATTCGATTTCCAATGTTGTGAATACCTC-3’ |
| **Cloning PCR^d^** |  |  |
| *aox1* promoter | 5’-TATGGTACCAGTAAATTCCAAACTAGCCATAGC-3’ | 5’-TATTTCGAAGAGAATAAATTGCCCGAGGCTAC-3’ |
| *nqo1* promoter | 5’-TATGGTACCCTACTTTGGGAGCTTGACCA | 5’-TATTTCGAAGAGGATCGTAATACCGAACGCTGA-3’ |
| Nrf2 ORF | 5’-TATTGATCAGGGTCGACAGCCATGGACTTGGAGT  TGCCACCGCCAGGACT-3’ | 5’-TATGCGGCCGCTCACAGTAGGAAGTTTTAGCAG  TAC-3’ |
