# Supplementary material for: Cytoprotective Nrf2 Pathway Is Induced In Chronically Txnrd 1-Deficient Hepatocytes
Source: PLoS One. 2009 Jul 7;4(7):e6158. doi: 10.1371/journal.pone.0006158 (PMC2703566; doi:10.1371/journal.pone.0006158)
Supplement: Supplementary file 4 [file pone.0006158.s002.doc]

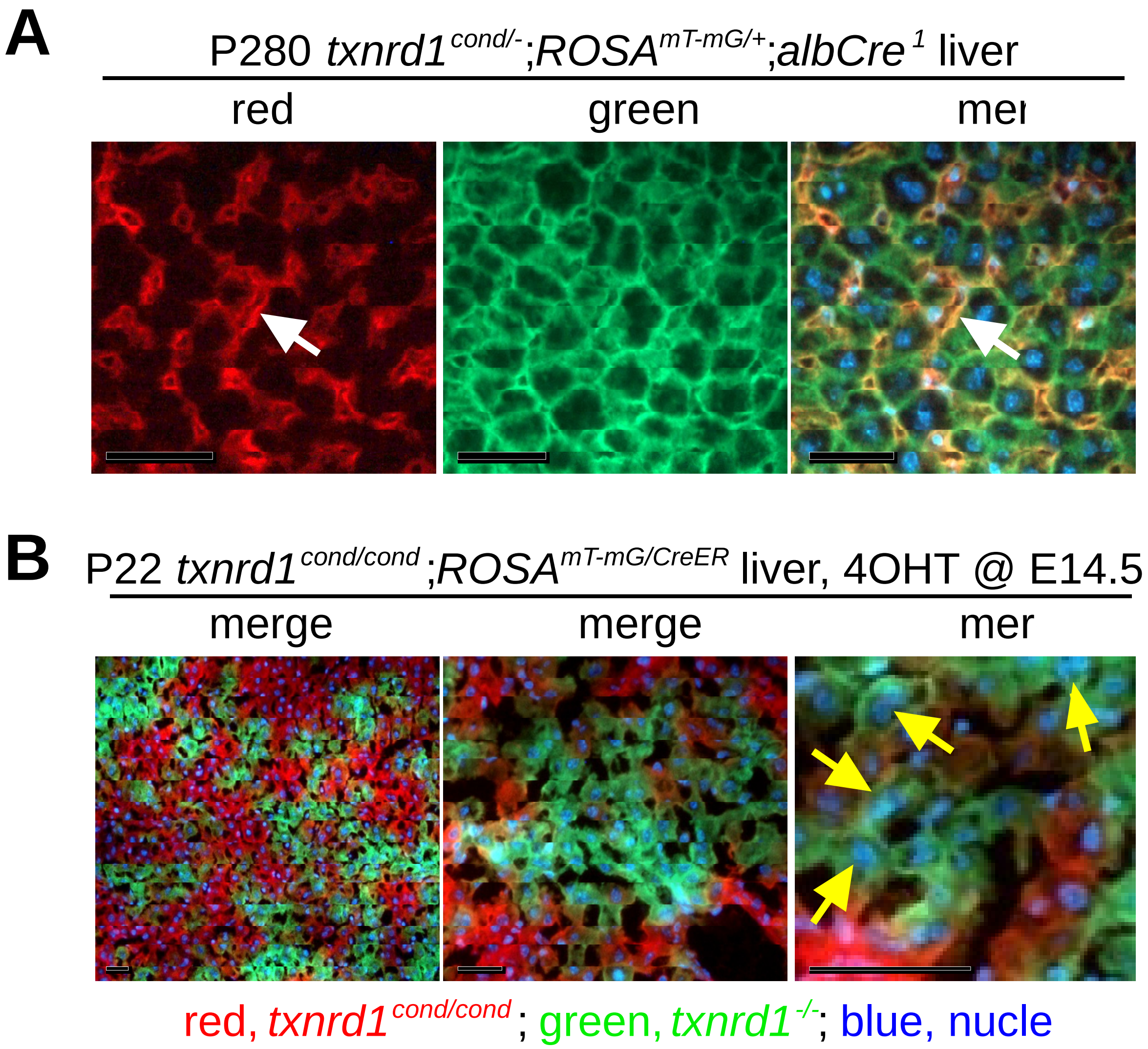
A
P280 txnrd1 cond/- ;ROSA mT-mG/+ ;albCre 1 liver
red
green
mer
B
P22 txnrd1 cond/cond ;ROSA mT-mG/CreER liver, 4OHT @ E14.5
merge
merge
mer
red, txnrd1 cond/cond ; green, txnrd1 -/- ; blue, nucle
